# A Systematic Review of the Influence of Overweight and Obesity across the Lifespan on Obstacle Crossing during Walking

**DOI:** 10.3390/ijerph20115931

**Published:** 2023-05-23

**Authors:** Matthias Chardon, Fabio A. Barbieri, Tiago Penedo, Paulo C. R. Santos, Nicolas Vuillerme

**Affiliations:** 1 Autonomie, Gérontologie, E-santé, Imagerie et Société (AGEIS), Université Grenoble Alpes, 38400 Grenoble, France; 2 Human Movement Research Laboratory (MOVI-LAB), Department of Physical Education, School of Sciences, São Paulo State University (Unesp), Bauru 15782, Brazil; 3 Department of Computer Science and Applied Mathematics, Weizmann Institute of Science, Rehovot 7632706, Israel; 4 Institut Universitaire de France, 75005 Paris, France

**Keywords:** obesity, overweight, obstacle crossing, gait, systematic review

## Abstract

This study aimed to systematically review and summarize the available data regarding the influence of overweight and obesity across the lifespan on obstacle crossing during walking. Four databases were systematically searched with no limitation on publication date following the Cochrane Handbook for Systematic Reviews and PRISMA guidelines. Only full-text English-language articles published in a peer-reviewed journal were eligible. They had to compare obstacle crossing during walking by overweight or obese individuals with individuals of normal body weight. Five studies were considered eligible. All the studies assessed kinematics; only one assessed kinetics, but none investigated muscle activity or obstacle contact. Compared to normal individuals crossing obstacles, overweight or obese individuals exhibited lower velocity, shorter step length, lower cadence, and less time spent in single-limb support. They also exhibited increased step width, more time spent in double support, and greater trailing leg ground force reaction and centre of mass acceleration. Overall, the small number of included studies did not allow us to draw any conclusions. However, being overweight or obese seems to have a potentially negative influence on the kinematics of gait parameters due to a tendency to trip, fall, and suffer severe fall-related injuries when negotiating obstacles on foot in real-life environments.

## 1. Introduction

The World Health Organization defines overweight (OW) and obesity (OB) as “abnormal or excessive fat accumulation that may impair health” [1]. OW and OB have become major health issues worldwide, and the prevalence of obesity continues to increase [2]. A 2016 World Health Organization report [1] estimated that 39% of the world population was considered OW and 13% was OB. Specific data for children and adolescents indicated that ~340 million 5–19-year-olds were OW or OB [1]. OW and OB are commonly characterised based on body mass index (BMI). For adults, a BMI of 25.0–29.9 kg/m^2^ is defined as OW, and a BMI of 30 kg/m^2^ or greater is defined as OB [3]. One emerging public health concern is that OW and OB are associated with several comorbidities that accentuate the risks of hospitalisation [4], sudden death [5] and functional impairments [6]. Among these functional impairments, static and dynamic balance and gait behaviours are particularly affected [7,8,9].

Molina-Garcia et al. [9] recently reported that OW and OB children and adolescents exhibited different gait behaviours than their normal-weight peers, including greater pelvis transversal plane motion, internal rotation/flexion, extension and abduction moments, power generation and absorption, knee abduction and adduction motion, and knee abduction and adduction moments. These biomechanical alterations are likely to increase the risk of injuries during the activities of daily living [10], musculoskeletal disorders [7,11], and fall risk [12] among individuals with OW and OB. However, although much has been reported about the influence of OW and OB on gait during unobstructed walking [13,14,15,16,17,18], little is known about how these conditions might impact gait under more challenging conditions, such as environments with obstacles [18,19,20,21,22].

Indeed, tripping during obstacle crossing represents one of the main causes (up to 53%) of falls among healthy older adults [23]. Over the past 25 years, obstacle crossing has been reported to reflect a greater risk of imbalance and trips and could lead to falls [24,25,26,27,28,29]. A possible explanation for the increased risk of tripping during obstacle crossing could be the increased neuromuscular demands during this activity compared to unobstructed walking [26,30,31]. Accordingly, given the functional limitations that OW and OB impose on the musculoskeletal system (see [9,10,32] for recent reviews), it seemed particularly interesting to evaluate whether and how OW and OB might impact obstacle crossing during walking. The present study aimed to systematically review and summarize the available data regarding the influence of overweight and obesity across the lifespan on obstacle crossing during walking.

## 2. Methods

### 2.1. Protocol and Registration

This systematic review’s protocol was registered with the International Prospective Register of Systematic Reviews (PROSPERO) (CRD#42021269949) and published in May 2022 [33]; it follows the recommendations of the Preferred Reporting Items for Systematic Reviews and Meta-Analyses (PRISMA) statement [34] (checklist available in the Supplementary Materials: Supplementary File S1) and the guidelines of the Cochrane Handbook for Systematic Reviews [35]. As this review was limited to publicly available materials, it did not require any ethical approval. Note that there was no deviation from the recently published protocol for systematic review [33].

### 2.2. Eligibility, Inclusion, and Exclusion Criteria

A population, intervention, comparison, outcome and study design (PICOS) tool was used to select the eligibility, inclusion and exclusion criteria for the studies reviewed. Two reviewers (MC and TP) independently included studies based on samples of OW or OB individuals (together defined as having a BMI ≥ 25 kg/m^2^ for adult studies or being at or above the 85th percentile of the Centers for Disease Control and Prevention’s Weight-for-recumbent-length Growth Charts for child studies [36]), excluding studies that selected participants with acute or overuse injuries or with neurological, musculoskeletal, or systemic diseases unrelated to OB comorbidities (population). We included studies addressing the influence of an obstacle-crossing task (intervention: obstacle-crossing while walking). Included articles must have compared obstacle-crossing during walking by OW or OB individuals to individuals of normal weight (NW) (BMI 18–24.9 kg/m^2^). No other walking tasks were considered (comparison). We included studies reporting kinematic, kinetic, or electromyographic parameters, and the following outcomes were also extracted (outcomes): gait spatial and temporal stride parameters (e.g., stride length and duration; and horizontal and vertical foot–obstacle clearance distances), kinetics (e.g., force outcomes such as momentum, work, power, and ground reaction force), muscle activation outcomes (e.g., amplitude, muscle onset, muscle activation duration, muscle synergy, mean frequency, and power density) and obstacle contact. Randomised controlled trials, non-randomised controlled trials, and non-randomised, non-controlled trials (study design) were all included. Accordingly, study design inclusion criteria required original articles to have been published in English in a peer-reviewed scientific journal. Finally, we excluded case reports, abstracts, editorials, letters to the editor, case studies, reviews, meta-analyses, theses, grey literature (annual, research, technical, or project reports), working papers, and government documents. The inclusion and exclusion criteria using the PICOS tool are described in Table 1.

### 2.3. Data Sources and Search Strategy

Team members developed and agreed upon the search strategy and selection criteria in line with the review questions. Following the recommendations in the PRISMA statement [34]⁠ and the guidelines in the Cochrane Handbook for Systematic Reviews [35], two reviewers (MC and TP) independently performed a systematic computerised literature search of PubMed, Web of Science, Scopus, and SportDiscus from their dates of inception to April 2022, and the search was repeated on February 2023. The search strategy targeted articles containing information relating to overweight or obesity and gait during obstacle-crossing tasks and all subsets of these terms. No filters were used, and the full combination of keywords for all the databases was:

(obes* OR overweight OR over-weight OR adipos* OR “body mass index” OR BMI) AND (“obstacle crossing” or “obstacle negotiation” or “obstacle avoidance” or “obstructed walking”). The references of included studies were not checked for potential citations. No experts in the field were contacted.

### 2.4. Study Selection

The same two reviewers (MC and TP) independently selected the studies, screened the titles, abstracts, and keywords identified via the search strategy, and applied the eligibility criteria. After this initial selection, full-length texts were subjected to the same procedure. In cases of disagreement, and if subsequent discussions between the two reviewers were inconclusive, a third reviewer (NV) was contacted to arbitrate until a consensus was found.

### 2.5. Data Extraction

In line with the PRISMA guidelines [34], the number of citations reviewed at each stage of the review was summarised in a flow diagram⁠. The same two reviewers (MC and TP) independently completed the data extraction process using a standardised data extraction form. The following four datasets were extracted from each article retrieved:

(1) Study characteristics: first author, title, year of publication, journal name, country, study design, mention of any adverse events occurring during the study, and funding;
(2) Sample description: sample size, age, sex, body weight, body height, body mass index, lower limb length, health status, fall status, and limb preference;
(3) Obstacle-crossing protocol: task requirement, instrumentation, data acquisition methodology, and gait parameters assessed;
(4) Main results obtained from gait measurement: spatial-temporal parameters, muscle activity, joint angle and moment, ground reaction force, and obstacle contacts.

### 2.6. Quality Assessment

The same two reviewers (MC and TP) independently performed a qualitative analysis of the selected studies [34]. To do this, they used a grid specifically developed by Galna and colleagues to assess the quality of the evidence contained within a systematic review investigating obstacle crossing by older adults [37]. Quality issues and risks of bias in the present systematic review centred on the internal validity, external validity, and reproducibility of the methods used in the articles retained. However, Galna’s grid [37] contains no items related to the study participants’ anthropometric characteristics. We thus modified the grid to allow us to address our review question: “How do overweight and obesity influence gait parameters during obstacle crossing across the lifespan?” (CRD#42021269949). The items of body mass and body mass index were added to the grid. The scoring system developed by Galna and colleagues [37] was used to quantify the quality of each of the studies retained and to assess the methodological strengths and weaknesses of those reviewed. Each question in our modified quality assessment [37] was scored as follows: 1 = assessment criterion met, 0 = assessment criterion not met, and 0.5 = lack of information or clarity on that criterion. Any discrepancies between the two reviewers’ findings were resolved by consensus. If disagreements persisted, a third reviewer (NV) was consulted to arbitrate a final decision.

### 2.7. Data Synthesis

Given the small number of included studies, the wide range of age of the participants included in these studies, and the variety of experimental protocols and reported outcomes, we were unable to conduct meta-analyses of the extracted data. As an alternative, we provided a narrative synthesis of the available data regarding the influence of overweight and obesity on kinematic (Section 3.6.1) and kinetic parameters (Section 3.6.2) related to executing the obstacle-crossing task.

## 3. Results

### 3.1. Study Selection

**Figure 1:**
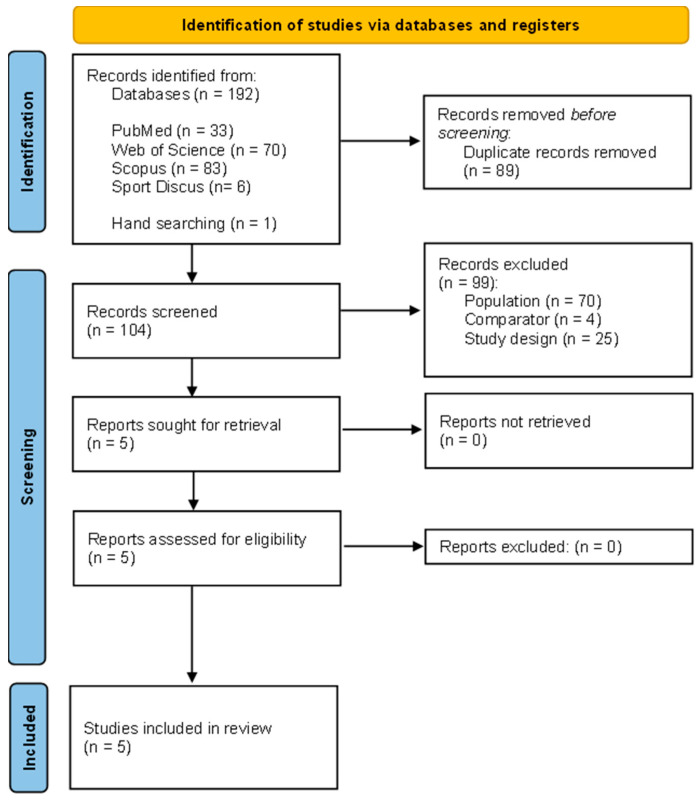
Flow diagram of the articles included in the review. The number of original articles is indicated at each stage of the search.

The study selection process is presented in Figure 1.

Our searches of PubMed, Web of Science, Scopus, and SportDiscus resulted in 33, 70, 83 and 6 records, respectively. One article was found via hand searching [21]. After duplicate removal (n = 89), 104 records remained. After screening titles, abstracts and keywords, five full texts were read to verify and confirm their eligibility [18,19,20,21,22]. All five met our eligibility criteria and were retained for review [18,19,20,21,22].

### 3.2. General Information about the Studies Included in the Systematic Review

Table 2 shows the basic information about the studies included in the systematic review.

### 3.3. Study Characteristics

Table 2 summarises the characteristics of the studies included in the review in order of publication.

The studies retained were published from 2012 to 2021, with one study each published in 2012 [19], 2014 [20], 2018 [21], 2019 [22] and 2021 [18].

The articles were published in four different journals: two in the *Journal of Musculoskeletal Neuronal Interactions* [18,22], and one each in the *Journal of Aging and Physical Activity* [21], *Research in Developmental Disabilities* [20] and the *American Journal of Physical Medicine and Rehabilitation* [19].

The studies originated from just two countries: four from the USA [18,19,20,22] and one from Portugal [21].

### 3.4. Quality Assessment

Table 3 summarises our quality assessment for each article retained [18,19,20,21,22].

The articles included adequately stated their objectives, provided an appropriate description of their participants (although three studies reported participants’ BMI but not their height or body mass [19,20,22]), described their inclusion and exclusion criteria, detailed their main findings, used appropriate methodologies to answer their research questions, and appropriately discussed their study results. The details provided were adequate to replicate their study. All five studies lacked detail and clarity on the clinical implications of their research [18,19,20,21,22].

#### Sample Characteristics

The basic demographic and anthropometric characteristics of each study’s participants are presented in Table 4.

Regarding sample size, a total of 132 OW/OB individuals and 82 NW individuals were included. Sample sizes ranged from 12 [19,20] to 54 [22] for OW/OB individuals and from 10 [20] to 27 [21] for NW individuals.

Regarding sex, two studies only included female participants [21,22], with 134 female participants in total (94 OW/OB; 40 NW). Three studies included male and female participants [18,19,20], totalling 34 males and 46 females. Two of these studies also reported the number of male and female participants in each BMI group, namely 9 OW/OB males and 17 OW/OB females vs. 12 NW males and 20 NW females [18,19]. No sex comparisons were made.

Regarding age, two studies only included children [19,20], with a total of 46 children (24 OW/OB individuals and 22 NW individuals). Children ranged from 4 to 13 years old across both studies [19,20].

Gill and Hung in 2012 reported their mean participant age as 8.58 ± 0.73 years for OW/OB individuals and 8.42 ± 1.00 years for NW individuals [19]. The mean participant age in the other study (including NW and OW/OB children) was 8.62 ± 0.93 years [20]. Three studies included adults [18,21,22], with a total of 168 participants (108 OW/OB; 60 NW), with mean ages ranging from 36.2 ± 12.8 [22] to 57.1 ± 4.7 [21] years for OW/OB individuals and from 33.2 ± 7.0 [22] to 58.2 ± 4.8 [21] years old for NW individuals. None of these three studies reported age inclusion criteria [18,21,22].

Considering anthropometry, two studies reported the heights and weights of their adult participants [18,21]. Heights ranged from 156.3 ± 4.5 cm [21] to 168.83 ± 8.69 cm [18] for OW/OB individuals and from 155.8 ± 5.2 cm [21] to 170.15 ± 8 cm [18] for NW individuals. Weights ranged from 73.1 ± 7.3 kg [21] to 120.58 ± 20.13 kg [18] for OW/OB individuals and from 58.4 ± 7.1 kg [21] to 69.36 ± 12.17 kg [18] for NW individuals. For the two studies involving children [19,20], BMIs were 21.41 ± 1.31 kg/m^2^ [19] and 21.85 ± 0.50 kg/m^2^ [20] for OW/OB individuals and 15.85 ± 0.68 kg/m^2^ for NW individuals [19,20]. For the three studies involving adults [18,21,22], BMIs ranged from 29.9 ± 2.5 kg/m^2^ [21] to 43.31 ± 4.24 kg/m^2^ [22] for OW/OB individuals and from 22.56 ± 1.61 kg/m^2^ [22] to 24.0 ± 2.0 kg/m^2^ [21] for NW individuals. Only Desrochers et al. (2021) reported participants’ heights and weights using fixed obstacle height [18].

Regarding obesity classification, four studies [18,19,20,22] used the Centers for Disease Control and Prevention classification [36,38], and the fifth [21] used a classification developed for Brochu et al.’s study [39] of post-menopausal women, where participants were considered OB above a BMI cut-off value of 27 kg/m^2^. In Desrochers et al.’s 2021 study involving adults [18], OB individuals had a BMI above 30 kg/m^2^. In another study, three OW/OB groups were established according to their BMI: OW/Class I OB (25 kg/m^2^ ≤ BMI < 35 kg/m^2^); Class II OB (35 kg/m^2^ ≤ BMI < 40 kg/m^2^); and Class III OB: (BMI < 40 kg/m^2^) [22].

In the two studies involving children aged 4–13 years old [19,20], participants at or above the 85th percentile and below the 95th percentile were classified as OW (approximately 17–25.2 kg/m^2^ and 17.2–26.2 kg/m^2^ for boys and girls, respectively), and those above the 95th percentile were classified as OB (approximately 17.8 to more than 25.2 kg/m^2^ and from 18 to more than 26.2 kg/m^2^ for boys and girls, respectively) [36].

### 3.5. Obstacle-Crossing Task

The obstacle-crossing tasks used in the retained studies are described in Table 5.

Regarding the walkway, four studies [18,19,20,22] reported fixed walkway lengths between 406 cm [19,20] and 1600 cm [22], and one [21] used a two-step protocol (left leg as the leading limb during obstacle crossing). Three studies placed their obstacles in the middle of their walkway [18,20,22], and one [19] did not report the obstacle’s location.

Concerning the starting point, studies’ starting points and walking distances before arriving at their obstacles ranged from 203 cm [20] to 800 cm [22] or two steps before the obstacle [21]. Gill and Hung’s 2012 study did not report the starting point’s distance from the obstacle [19].

For the obstacle conditions and experimental conditions, all five studies performed an overground gait task and obstacle-crossing tasks [18,19,20,21,22]. Four studies used baseline and final overground walking trials [18,19,20,22]; three studies executed five consecutive overground walking trials before and after their obstacle-crossing tasks [18,20,22] Desrochers et al., 2021 used a single overground walking trial before and after the obstacle-crossing task [19]. In the last study, participants performed five trials using the two-step protocol without the presence of the obstacle [21].

Regarding obstacle height and shape, Silva et al., 2018 [21] used an obstacle height that varied according to the participant’s leg length (30% of leg length), with obstacle heights of 22.2 ± 0.9 cm for non-OB participants and 22.1 ± 1 cm for OB participants. The obstacle’s shape was not described, however. Four studies [18,19,20,22] used three fixed obstacle heights: two [19,20] used small (4 cm), medium (11 cm), and high (16 cm) obstacles; two [18,22] used different respective small (4 cm), medium (8 cm), and high (16 cm) obstacles. All four [18,19,20,22] used a wooden dowel suspended across the walkway and inserted into holes in two wooden towers as their obstacle.

As concerns instructions, in all five studies, participants were instructed to walk at a self-selected speed and cross an obstacle. In the study by Gill (2019) [22], participants walked barefoot; in the other four studies [18,19,20,22], footwear was not specified. The obstacle’s presence on the walkway was expected in all five studies. Participants executed 5 consecutive trials for each obstacle condition [18,19,20,21,22]. The mean [18,19,20,21,22] or mean and SD were used to calculate coefficients of variation using the following equation: the mean/SD [22] of the 5 trials was used for data analysis. Three studies reported that the obstacle condition order was counterbalanced [18,19] or randomised [22] across individuals; Gill and Hun, 2014 did not mention any obstacle condition order [20].

Regarding practice and familiarisation, two studies [20,21] reported that the participants were allowed to practice before the experimental trials, with one permitting three practice trials [20] and the other allotting a 10 min familiarisation period [21].

Table 6 reports the categories of parameters related to the performance of the obstacle-crossing tasks assessed in each of the retained studies, including the parameters measured during obstacle crossing, and the data acquisition methods for kinematic and kinetic gait parameters related to the performance of the obstacle-crossing tasks.

All five studies assessed kinematic parameters [18,19,20,21,22], but only one assessed both the kinematic and kinetic parameters related to executing the obstacle-crossing task [20]. None of the studies assessed muscular activity or obstacle contacts.

Regarding kinematic parameters, the kinematic measurement tools used were Protokinetics LLC’s 610 cm long × 89 cm wide gait carpet [22], GAITRite’s 488 cm long × 61 cm wide carpet [18] and two Footscan platforms (100 × 40 cm) [21]. Two studies used the Vicon Nexus Model motion capture system [19,20].

The only kinematic parameter investigated by two studies was gait velocity during obstacle crossing [18,22]. The following 63 kinematic parameters were only assessed in one study each:

- Step length, width, cadence, and single- and double-limb support phases [18];
- Stance and swing time and their coefficients of variation, and the coefficient of variation of velocity [22];
- Time from foot lift to maximum knee height and from maximum knee height to foot contact, maximum knee height (plus sagittal knee and ankle angles at maximum knee height), and ankle angle at foot contact [19];
- Leading- and trailing-leg toe clearance, hip and knee angles at maximum knee height in the sagittal and frontal planes, COM (centre of mass) anterior and posterior, and medial, lateral and vertical acceleration at the leading and trailing legs’ maximum knee height during crossing [20];
- Relative foot temporal data at initial and final foot contact and duration of contact of the HL (lateral heel), HM (medial heel), MF (midfoot), M1-5 (metatarsal areas), T2-5 (toes), and T1 (hallux) areas of the leading and trailing legs [21].

Regarding kinetic parameters, Gill and Hung, 2014 [20] assessed six kinetic parameters using two AMTI OR6-6 force platforms (46 cm × 50 cm): leading and trailing leg normalised anterior and posterior; and medial, lateral, and vertical ground reaction force at maximum knee height of the contralateral leg during obstacle crossing [20].

### 3.6. Influence of Overweight/Obesity on Obstacle Crossing during Walking

#### 3.6.1. Kinematic Parameters

Table 7 reports the influence of OW/OB on kinematic parameters during obstacle crossing. 

Regarding gait velocity, both studies that investigated gait velocity [18,22] reported that the OB (>30 BMI), [18] OW/Class I OB (25 kg/m^2^ ≤ BMI < 35 kg/m^2^), Class II OB (35 kg/m^2^ ≤ BMI < 40 kg/m^2^), and Class III OB (BMI > 40 kg/m^2^) [22] groups’ step-crossing velocities were slower than those of the NOB (non-OW/OB) group (≥18.5 kg/m^2^ and <25 kg/m^2^) [18,22]. Results also showed that Class II OB and Class III OB participants had a slower step-crossing velocity than Class I OB participants [22].

Being OB led to a significantly lower crossing step length [18].
The OB group showed a significantly lower cadence [18].
OB individuals spent less time in single limb support [18].
The OB group had a greater step width [18].
OB individuals spent more time in the double-limb support phase [18].
The OW/Class I OB group spent more time in stance than the NOB and Class II OB groups [22].
The OW/Class I OB, Class II OB, and Class III OB groups had lower swing times during crossing [22].

OB individuals showed faster COM anterior/posterior (A/P) acceleration when crossing the low obstacle (4 cm) during both-leg crossing [20]. Additionally, when the trailing leg crossed the obstacle, COM A/P acceleration was faster on low obstacles than on medium and high obstacles, but only for the OB group [20].

Regarding leg motion during crossing, OB individuals took longer from foot lift to maximum knee height and less time from maximum knee height to foot contact in the high obstacle (16 cm) condition, with obstacle conditions having no effects on maximum knee height [19].

The OB group had a smaller ankle angle at foot contact during the low obstacle (4 cm) condition [19].

Regarding vertical clearance, OB individuals showed greater trailing-leg toe clearance in the low (4 cm) obstacle condition than in the high one (16 cm), whereas the NOB group showed greater trailing-toe clearance in the high obstacle condition than in the low one. No influence of OB was observed for leading-leg toe clearance [19].

The OB group’s leading-leg frontal hip angle at maximum knee height was greater in high (16 cm) obstacle conditions than in medium (11 cm) and low (4 cm) ones. No effects were observed for the NOB group [20].

The OB group showed a greater trailing leg sagittal knee angle at maximum knee height than the NOB group for the low and high obstacle conditions, but no effect was observed for the medium obstacle condition [20].

Regarding temporal foot data, there were no differences between the OB and NOB groups regarding their relative temporal data at initial and final contact or the contact duration of the plantar areas assessed (HL; HM; MF; M1; M2; M3; M4; M5; T2-5; T1) [21].

Regarding the coefficient of variation of gait velocity, no differences were found between the OW/Class I OB and NOB groups [22]. The Class II OB and Class IIB OB groups had a greater coefficient of variation of velocity than the NOB and OW/Class I OB groups [22].

#### 3.6.2. Kinetic Parameters

Table 7 reports the influence of OW/OB on kinetic parameters during obstacle crossing.

Gill and Hung, 2014 investigated kinetic parameters during obstacle crossing [20].

Regarding trailing-leg normalised ground reaction forces (GRF), the OB group showed a greater trailing-leg normalised A/P GRF during the low obstacle condition (4 cm) than in the medium (11 cm) and high conditions (16 cm) [20]. The NOB group showed greater trailing-leg normalised A/P GRF in high obstacle conditions than in medium and low ones [20]. Trailing-leg normalised medial/lateral (M/L) GRF was only greater in the high obstacle condition than in the medium one for the OB group [20]. In the OB group, trailing-leg normalised vertical GRF was greater in the high obstacle condition than in the medium one, whereas for the NOB group, trailing-leg normalised vertical GRF was greater during the medium obstacle condition than during the low one.

For leading-leg normalised GRF, no effects on the OB group were observed for any GRF [20].

### 3.7. Synthesis

Five studies were considered eligible and were retained [18,19,20,21,22]. Overall, these studies were evaluated as “good” despite their lack ofs detail and clarity on the clinical implications of their research. Among these 5 studies, 2 included children [19,20], and 3 included adults [18,21,22]. One study used an obstacle height that varied according to the participant’s leg length (30% of leg length) [21], while four studies used three fixed obstacle heights [18,19,20,22]. All the studies [18,19,20,21,22] included single-task conditions, used self-selected walking speed instruction, and had the same number of experimental sessions (n = 1) and trials per obstacle crossing conditions (n = 5). Finally, all the studies assessed kinematics [18,19,20,21,22], one assessed kinetics [20], and none investigated muscle activity or obstacle contact. Compared to normal individuals crossing obstacles, overweight or obese individuals exhibited lower velocity [18,22], shorter step length [18], lower cadence [18] and less time spent in single limb support [18]. They also exhibited increased step width [18], more time spent in double support [18], and greater trailing leg ground force reaction [20] and center of mass acceleration [20]. Furthermore, these observed between-group differences were also dependent on obstacle height [19,20].

## 4. Discussion

This work aimed to systematically review and summarize all the available biomechanical-parameter data regarding the influence of OW and OB across the lifespan on obstacle-crossing tasks during walking tests. Our broad search and selection retained five studies for evaluation. Two studies involved children [19,20], and three involved adults [18,21,22], for a total of 82 NW individuals and 132 OW/OB individuals. Their performances in the different obstacle-crossing tasks were assessed using a wide variety of kinematic and kinetic parameters. Overall, results suggested that compared to NW individuals, OW and OB individuals walked more slowly and with a lower cadence, shorter step length, shorter single-limb support duration, shorter swing time, longer leg-raising and shorter leg-dropping times, and a smaller ankle angle at foot contact.

### 4.1. Influence of Overweight/Obesity on Kinematic Parameters during Obstacle Crossing

The most consistent between-group difference (NW vs. OB) in obstacle-crossing performance was the lower gait velocity in OB adults reported in two studies comparing them to NW adults [18,22]. One of those two studies [22] reported that adults in the OW/Class I OB (≥25 kg/m^2^ and <35 kg/m^2^) group had faster gait velocities and less variable velocities than those in the Class II OB (≥35 kg/m^2^ and <40 kg/m^2^) or Class III OB (>40 kg/m^2^) groups. In addition to slower velocity, Desrochers et al. (2021) [18] reported that OB adults had a greater step width and double-limb support duration and shorter step length, single-limb support duration, and cadence than NW individuals. This study’s authors [18] proposed the following interpretation for their results: “These gait changes likely represent adaptive behaviors in the face of unstable postural control, where individuals with obesity increase their base of support for a greater amount of time relative to a single gait cycle” [18]. It is noteworthy that these authors [18] further speculated that the shorter single-limb support duration measured in OB adults might increase the likelihood of tripping as they cross the obstacle more quickly and with less clearance.

All five studies assessed spatial outcome measures of gait [18,19,20,21,22]. Ankle angle at landing after obstacle crossing differed significantly between NW and OB children in the low obstacle condition (4 cm) [19]. NW children tended to land in a neutral ankle-angle position (around 90°, corresponding to a flat-foot landing strategy), whereas OB children were more dorsiflexed (<90°, corresponding to a heel–toe landing strategy) [19]. The study authors [19] suggested that NW children may have used a flat-foot strategy during obstacle crossing because it was the most useful for increasing landing stability, as a heel–toe strategy was less stable. Walking impairments could lead to falls and injuries in OB children [40]. Note that the heel–toe landing strategy reported in OW/OB children was not observed in OB post-menopausal women, who exhibited similar relative initial, final, and duration of contact for all ten-foot areas and foot total-contact duration to NW post-menopausal women [21]. NW and OB children had different trailing-vertical-toe clearances depending on the obstacle condition [20]. Interestingly, OB children had a higher clearance over low obstacles than over high ones, whereas NW children had a higher clearance over high obstacles than over low ones. This study’s authors [20] suggested that: “overweight/obese children’s difficulty with motor planning may have contributed to the use of a less effective strategy when crossing high obstacles. (…) differences in motor skills and strategies used during obstacle crossing can increase the risk of injury; less toe clearance on high obstacles increases the chance of tripping and falling” ([20], p. 51).

All five studies assessed temporal outcome measures of gait [18,19,20,21,22]. Compared with NW children, OB children took longer to reach maximum knee height and less time to move from maximum knee height to foot contact in the high (16 cm) obstacle condition [19]. These authors suggested that this behaviour could stem from OB children’s relatively limited capacity to accelerate lower-leg segments due to the relatively higher effort and energy they expend compared to NW children [19]. They also suggested that the longer time OB children required to lift their knees (with no difference in knee height) compared to NW children may be due to musculoskeletal difficulties [19], especially due to OB children’s heavier lower limbs [41]. This interpretation is supported by overground level-walking data showing that even when over-activating ankle muscles, obesity-typical spatiotemporal changes in gait were observed [42]. However, it is important to mention that this interpretation remains speculative since no studies have directly measured muscle activation during obstacle crossing to compare OB and/or OW individuals to NW ones. This point reinforces the relevance and necessity of future studies investigating physiological and mechanical mechanisms related to impaired gait control in OW/OB individuals. Interestingly, significant differences in COM anterior/posterior acceleration between NW and OB children were only observed in the low obstacle condition (4 cm), where COM acceleration at the maximum knee height of both legs during crossing was higher among OB children [20]. The authors suggested that this observation could reflect the difficulties OB children had in controlling forward acceleration [20] when crossing a low obstacle.

### 4.2. Influence of Overweight/Obesity on Kinetic Parameters during Obstacle Crossing

The impaired gait control and stability observed during obstacle crossing due to OW/OB were reiterated in the kinetics results. NW and OB children exhibited significant differences in trailing-leg ground force reactions. Compared to the NW group, in the anterior/posterior direction, OB children had a higher normalised ground reaction force in low obstacle conditions than in medium or high obstacle conditions, and in the vertical and medial/lateral directions, their ground forces were significantly higher in the high obstacle condition than in the medium one (Table 7, [20]). Among NW children, differences in obstacle conditions were only observed in the anterior/posterior direction (a higher value on the high obstacle condition than on the medium or low ones) and the vertical direction (a higher value on the medium obstacle condition than on the low one) [20]. This study’s authors [20] suggested that OB children’s balance issues [43] and musculoskeletal disorders [41] could have been exacerbated by their larger hip abduction angles and higher vertical and medial/lateral ground reaction forces, especially on high obstacles. They also suggested that OB children’s efforts to maintain stability could have been more difficult due to changes in hip abduction angles and the vertical and medial/lateral ground reaction forces on high obstacles [44].

### 4.3. Limitations and Strengths

The present systematic review had some limitations, most notable of which was the low number of studies (n = 5) [18,19,20,21,22] meeting our selection criteria and their heterogeneity, including differences in the sample populations, experimental procedures, and gait outcomes. Indeed, participants’ age groups differed widely between the 5 included studies; two included children aged 4–13 years old [19,20], while three included adults [18,21,22] aged 36.16 ± 12.76 [22] and 57.1 ± 4.7 years old [21], resulting in small sample sizes within each age category (with a total of 24 OW/OB child participants vs. 22 NW child participants [19,20] and 108 OW/OB adult participants vs. 60 NW adult participants) [18,21,22]. However, the large age range of the individuals can also be regarded as a strength of the present review that allowed us to summarize the available data regarding the influence of overweight and obesity across the lifespan on obstacle crossing during walking. Another limitation is that none of the studies that included both male and female participants [18,19,20] evaluated the influence of sex on gait outcomes, although previous studies have reported sex differences in gait patterns during unobstructed level walking [45]. It is also noteworthy that although all the studies included had assessed BMI-group-related differences in gait outcomes during obstacle crossing [18,19,20,21,22], none had specifically reported the possible influence of body mass and body height, i.e., the two constitutive anthropometric parameters of BMI. This observation is all the more important given that 4 of the 5 included studies [18,19,20,22] used three fixed obstacle heights (low, medium and high obstacles, measuring from 4 cm to 16 cm), i.e., not personalised to each participant’s bodily dimensions. Note that, in an effort to scale the difficulty of an obstacle crossing task to each participant’s bodily dimensions, one of the five included studies [21] used an obstacle height that reflected a percentage of each individual’s leg length (i.e., 30%, representing approximately 22 cm for NW and OW/OB individuals) [21]. In addition to the above-mentioned differences in sample populations and experimental procedures, the present systematic review also revealed the heterogeneity in gait outcomes in each of the included articles, which further hampered the comparison of published results. Interestingly, although it is likely that greater toe–obstacle clearance may help decrease the risk of tripping over an obstacle (e.g., [31,46,47,48,49]), this parameter (namely leading-leg toe clearance and trailing-leg toe clearance) was reported in only one of the five included studies [20]. Furthermore, participants’ obstacle contacts (errors) during the execution of the obstacle-crossing task were not reported in any of the 5 included studies.

### 4.4. Practical Implications and Future Directions

Given the results, limitations, and strengths of the present review, several practical implications can be considered. It is presumable that the performance of an obstacle-crossing task depends on the participant’s height (and/or their lower limb length) and the height of the obstacle [31]. Accordingly, a description of the participant’s height and leg length should be reported in a systematic way. As above-mentioned, in addition, or in combination with the influence of anthropometric parameters, future studies should investigate how sex could affect the performance of obstacle-crossing tasks in normal weight and OW/OB individuals. Finally, we believe that obstacle contacts and foot clearances should be assessed and reported in a systematic way. We believe, indeed, that these parameters could be used to identify OW/OB individuals at risk of falling and to evaluate the outcomes of interventions aimed at fall prevention.

## 5. Conclusions

Despite the small number of studies included, the present findings suggest that overweight and obesity do indeed affect kinematic and kinetic gait parameters during obstacle-crossing tasks executed by both children [19,20] and adults [18,21,22]. At this point, however, the kinematic parameters assessed in each study were diverse; only one of these five studies investigated kinetics, and none assessed muscular parameters or obstacle contacts. These could be appropriate research elements for future studies, as could investigating the influence of specific anthropometric parameters. Finally, these findings should be interpreted with caution as no overarching or generalizable conclusions could be drawn regarding the influence of overweight and obesity across the lifespan on obstacle crossing during walking.

## Figures and Tables

**Table 1:** Eligibility criteria of the included studies using PICOS.

	Inclusion Criteria	Exclusion Criteria
Population	Overweight or obese individuals	Acute or overuse injuries or with neurological, musculoskeletal or systemic diseases unrelated to OB comorbidities
Intervention	Obstacle-crossing task during walking	Obstacle avoidance during a non-walking task Walking task without obstacle crossing
Comparator	Healthy normal-weight individuals	Non-healthy normal-weight individuals
Outcomes	Kinematic, kinetic, and electromyographic	None
Study design	Randomised controlled trials, non-randomised controlled trials, and non-randomised, non-controlled trials. Published in English in a peer-reviewed journal	Case reports, abstracts, editorials, letters to the editor, case studies, reviews, meta-analyses, theses, grey literature (annual, research, technical, or project reports), working papers, and government documents

**Table 2:** Studies included in the review in order of publication.

Author	Gill and Hung [19]	Gill and Hung [20]	Silva et al. [21]	Gill [22]	Desrochers et al. [18]
Publication year	2012	2014	2018	2019	2021
Country	USA	USA	Portugal	USA	USA
Title	Influence of weight classification on children stepping over obstacles	Effects of overweight and obese body mass on motor planning and motor skills during obstacle crossing in children	Foot rollover temporal parameters during walking straight ahead and stepping over obstacles: obese and non-obese post-menopausal women	Effects of obesity class on flat ground walking and obstacle negotiation	Association between the Functional Gait Assessment and spatiotemporal gait parameters in individuals with obesity compared to normal-weight controls: A proof-of-concept study
Journal	*American Journal of Physical Medicine and Rehabilitation*	*Research in Developmental Disabilities*	*Journal of Aging and Physical Activity*	*Journal of Musculoskeletal Neuronal Interactions*	*Journal of Musculoskeletal Neuronal Interactions*
Study design	Observational cross-sectional	Observational cross-sectional	Observational cross-sectional	Observational cross-sectional	Observational cross-sectional, proof-of-concept
Main objective	To examine how weight classification relates to children’s ability to meet a task constraint: crossing obstacles of various heights.	To examine whether body mass index would influence the ability of 4- to 13-year-olds to plan and coordinate their movements to cross obstacles of various heights.	To explore the potential differences between walking straight ahead and walking stepping over obstacles for OB and NOB post-menopausal women.	To investigate how increasing obesity classes affected gait and gait variability in adults.	To determine how to capture gait and balance impairments in adults with obesity using an inexpensive method as a proof-of-concept for possible future validation.
Main Findings	During obstacle crossing, children who were overweight or obese took longer to cross obstacles and had a dorsiflexed ankle position when landing. We also found that children demonstrated high variability in ankle position when crossing medium obstacles and during the final baseline trials.	Differences in motor planning and motor skills between normal weight and overweight/obese children during obstacle crossing may reflect movement patterns evident during early skill acquisition in which children attempt to freeze degrees of freedom, exhibit difficulty planning and controlling their movements with excess adiposity, or use unknown mechanisms responsible for motor planning and motor skill abilities	Significant differences were found in temporal characteristics of foot rollover during walking straight ahead and stepping over obstacles in both groups, with most of these differences being common for both OB and NOB subjects.	Increases in classes of obesity are associated with more difficulties with spatiotemporal gait and gait variability. Most importantly, there were few differences between Class II and Class III obesity.	Poorer FGA scores in the obese group were associated with slowing of gait when encountering obstacles but not during flat over-ground walking. Further, the presence of obstacles during gait tasks may be helpful in revealing meaningful gait impairments in obesity and other populations.
Funding	No funding information reported.	Boston University start-up funds.	European Investment Funds via FEDER/COMPETE/POCI—Operational Competitiveness and Internationalisation Programme, under project POCI-01-0145-FEDER-006958 and National Funds via FCT—Portuguese Foundation for Science and Technology, under project UID/AGR/04033/2013	R03 AR066344-01 A1 (Gill, PI).	NIH R03 AR066344-01A1 (Gill, PI).

Note: FGA: Functional Gait Assessment; NOB: non-obese; OB: obese.

**Table 3:** Methodological quality appraisal results adapted from the grid developed by Galna et al. [36].

Question	Scoring Criteria	Gill and Hung [19]	Gill and Hung [20]	Silva et al. [21]	Gill [22]	Desrochers et al. [18]	Average
1. Research aims or questions stated clearly	Y = 1; L = 0.5; N = 0	1	1	1	1	1	1
2. Participants detailed	Number	1	1	1	1	1	1
	Age	1	1	1	1	1	1
	Sex	1	1	1	1	1	1
	Height	0	0	1	0	1	0.4
	Body mass	0	0	1	0	1	0.4
	Body mass index	1	1	1	1	1	1
	Subtotal	0.67	0.67	1	0.67	1	0.8
3. Recruitment and sampling methods described	Y = 1; L = 0.5; N = 0	1	1	1	1	1	1
4. Inclusion and exclusion criteria detailed	Y = 1; L = 0.5; N = 0	1	1	1	1	0.5	0.9
5. Covariates controlled for	Age	1	1	1	1	1	1
	Sex	0	0	1	1	0	0.4
	Height	0	0.5	1	0	0	0.3
	Body mass	0	0	0	0	0	0
	Body mass index	1	1	1	1	1	1
	Limb asymmetries	0	0	1	0	1	0.4
	Strength	0	0	0	0	0	0
	Stride/step Speed	1	1	1	1	0	0.8
	Subtotal	0.38	0.44	0.88	0.5	0.38	0.52
6. Key outcome variables clearly described	Y = 1; L = 0.5; N = 0	1	1	1	1	1	1
7. Adequate methodology ables study replication	Participants	0.5	0.5	1	0.5	1	0.7
	Equipment	1	1	1	1	1	1
	Procedure	1	1	1	1	1	1
	Processing	1	1	1	1	1	1
	Statistics	1	1	1	1	1	1
	Subtotal	0.9	0.9	1	0.9	1	0.94
8. Methodology able to answer the research question	Participants	1	1	1	1	1	1
	Equipment	1	1	1	1	1	1
	Procedure	1	1	1	1	1	1
	Processing	1	1	1	1	1	1
	Statistics	1	1	1	1	1	1
	Subtotal	1	1	1	1	1	1
9. Reliability of the methodology is stated	Y = 1, N = 0	0	0	0	0	0	0
10. Internal validity of the methodology was stated	Y = 1, N = 0	0	0	0	0	0	0
11 Research questions were answered adequately in the discussion	Y = 1, N = 0	1	1	1	1	1	1
12. Key findings were supported by the results	Y = 1, N = 0	1	1	1	1	1	1
13. Key findings were interpreted logically, supported by references	Y = 1, N = 0	1	1	1	1	1	1
14. Clinical implications were stated	Y = 1; L = 0.5; N = 0	0	0.5	0	0	0	0.1
Study average		0.69	0.71	0.85	0.72	0.75	0.74

Y = yes; L = lacking detail or clarity; N = no. A score of 1 indicated high-quality research, and 0 indicated lower quality.

**Table 4:** Basic demographic and anthropometric characteristics of the participants in each study.

Author	Gill and Hung [19]	Gill and Hung [20]	Silva et al. [21]	Gill [22]	Desrochers et al. [18]
Number of groups (n)	2:	2:	2:	4:	2:
	Normal BMI (between 5th and 85th percentile). ^(C)^	Normal weight (between 5th and 85th percentile). ^(C)^	Non-OB (<27 kg/m^2^).	Normal BMI (≥18.5 kg/m^2^ and <25 kg/m^2^).	Normal BMI (≥19 kg/m^2^ and ≤25 kg/m^2^).
	High BMI (children at or above 85th percentile and below 95th percentile were classified as OW; those above 95th percentile were classified as OB). ^(C)^	OW/OB (children at or above 85th percentile and below 95th percentile were classified as OW; those above 95th percentile were classified as OB). ^(C)^	OB ^(Z)^ (>27 kg/m^2^).	OW/Class I OB ^(C)^ (≥25 kg/m^2^ and <35 kg/m^2^).	OB BMI (≥30 kg/m^2^). ^(C)^
				Class II OB ^(C)^ (≥35 kg/m^2^ and <40 kg/m^2^).	
				Class III OB ^(C)^ (>40 kg/m^2^).	
Number of participants (n)	Non-OB: 10	Non-OB: 10	Non-OB: 27	Normal BMI: 13	Non-OB: 20
				OW/Class I OB: 18	
	OW/OB: 12	OW/OB: 12	OB: 40	Class II OB: 16	OB: 14
				Class III OB: 20	
Sex (F: female; M: male) (n)	Non-OB: F:6; M: 6	All participants: F: 9; M: 13	Non-OB: F: 27	Normal BMI: F: 13	Non-OB: F: 14; M: 6
				OW/Class I OB: F: 18	
	OW/OB: F:5; M: 7		OB: F: 40	Class II OB: F: 16	OB: F: 12; M: 2
				Class III OB: F: 20	
Exclusion criteria	Not reported.	Not reported.	(1) Diabetes and/or signs associated with neuropathy, (2) acute foot pain and deformities, (3) severe lower extremity trauma, and (4) coordination problems resulting from eye disorders. Scheduled to undergo knee surgery, having no significant cardiovascular, musculoskeletal, vestibular or other neurological disorders. These criteria were confirmed via participant reports and investigators’ observations.	Scheduled to undergo knee surgery, having no significant cardiovascular, musculoskeletal, vestibular or other neurological disorders. These criteria were confirmed via participant reports and investigators’ observations.	Not reported.
Inclusion criteria	Being free of intellectual impairment or physical conditions that precluded independent walking based on parents’ reports and investigators’ observations and being 4–13 years old.	Having normal cognitive abilities, no known physical conditions that would preclude independent walking, and being 4–13 years old.	Not reported.	All participants could walk without the aid of an assistive device.	All participants were free of neurological difficulties, had normal or corrected-to-normal vision, and could walk without assistive devices.
Age, mean (SD) (Range), years	Non-OB: 8.42 (1.00) (4.5–13).	All participants: 8.62 (0.93) (not reported).	Non-OB: 58.2 (4.8) (not reported).	Normal BMI: 33.2 (7.04) (not reported).	Non-OB: 45.55 (8.77) (35–64).
				OW/Class I OB: 36.16 (12.76) (not reported).	
	OW/OB: 8.58 (0.73) (6–13).		OB: 57.1 (4.7) (not reported).	Class II OB: 41.17 (5.89) (not reported)	OB: 50.36 (10.97) (35–66).
				Class III OB: 42.72 (11.43) (not reported).	
Height, mean (SD) (Range), cm	Not reported.	Not reported.	Non-OB: 155.8 (5.2) (not reported).	Not reported.	Non-OB: 170.15 (8) (150–180). ^(X)^
			OB: 156.3 (4.5) (not reported).		OB: 168.83 (8.69) (155–184). ^(X)^
Body mass, mean (SD) (Range), kg	Not reported.	Not reported.	Non-OB: 58.4 (7.1) (not reported).	Not reported.	Non-OB: 69.36 (12.17) (47.20–86.20). ^(X)^
			OB: 73.1 (7.3) (not reported).		OB: 120.58 (20.13) (99.20–173.01). ^(X)^
BMI, mean (SD) (Range), kg/m^2^	Non-OB: 15.85 (0.68) (11.65–18.35). ^(C)^	Non-OB: 15.85 (0.68) (not reported). ^(C)^	Non-OB: 24.0 (2.0) (not reported).	Normal BMI: 22.56 (1.61) (not reported).	Non-OB: 23.77 (2.53) (18.59–27.55). ^(C)^
				OW/Class I OB: 29.36 (3.19) (not reported). ^(C)^	
	OW/OB: 21.41 (1.31) (16.67–31.23). ^(C)^	OW/OB: 21.85 (0.50) (not reported). ^(C)^	OW/OB: 29.9 (2.5) (not reported).	Class II OB: 37.78 (1.42) (not reported). ^(C)^	OB: 40.95 (5.46) (35.3–52.64). ^(C)^
				Class III OB: 44.31 (4.24) (not reported). ^(C)^	

BMI: body mass index. Class I obesity: 30 kg/m^2^ ≤ BMI < 35 kg/m^2^; Class II obesity: 35 kg/m^2^ ≤ BMI < 40 kg/m^2^; Class III obesity: BMI > 40 kg/m^2^). ^(C)^ indicates an OW and OB classification according to the Centers for Disease Control and Prevention [38]; ^(Z)^ indicates that OB levels were determined using the cut-off value of 27 kg/m^2^: Brochu et al., 2008. “Contribution of the Lean Body Mass to Insulin Resistance in Postmenopausal Women with Visceral Obesity: A Monet Study” [39]; ^(X)^ indicates that the value was calculated by the reviewers. Non-OB: non-overweight/obese individuals; OW: overweight individuals.

**Table 5:** Description of the obstacle-crossing tasks used in the retained studies.

Author	Gill & Hung [19]	Gill & Hung [20]	Silva et al. [21]	Gill [22]	Desrochers et al. [18]
Task	Participants first walked once along a 406-cm-long walkway. In balance order, they walked and stepped over low, medium, and high obstacles.	Children walked along a 406-cm-long walkway and crossed low, medium, and high obstacles.	Walking straight ahead with or without stepping over an obstacle whose height was 30% of the leg length in a two-step protocol.	Participants walked down a 1600 cm walkway with a gait carpet (610 cm long × 89 cm wide) in the centre and crossed 3 obstacles of low, medium, and high height placed halfway down the path.	Participants walked along the GAITRite (488 cm long × 61 cm wide) walkway under five conditions.
Obstacle	A wooden dowel inserted into two 25-cm-high wooden towers at 4 cm (low obstacle), 11 cm (medium obstacle), and 16 cm (high obstacle).	A wooden dowel inserted into two 25-cm-high wooden towers at 4 cm (low obstacle), 11 cm (medium obstacle), and 16 cm (high obstacle).	Not reported.	The obstacles were created using a wooden dowel (121 cm long) and two rectangular towers (9 cm × 10 cm × 22 cm) with holes drilled at 4 cm, 8 cm, and 16 cm (low, medium, and high).	The obstacle was a wooden dowel suspended across the walkway that was inserted into holes in two wooden towers. Participants encountered small (4 cm), medium (8 cm), or large (16 cm) obstacles.
Expected/Unexpected	Expected.	Expected.	Expected.	Expected.	Expected.
Starting point	Not reported.	203 cm from the obstacle.	Two-step protocol with the left leg as the leading limb during obstacle crossing.	800 cm from the obstacle.	244 cm from the obstacle.
Number of sessions	1.	1.	1.	1.	1.
Number of conditions	5: No obstacle = flat surface without obstacle (baseline initial and final trials). Low obstacle height (4 cm). Medium obstacle height (11 cm). High obstacle height (16 cm).	5: No obstacle = flat surface without obstacle (baseline initial and final trial). Low obstacle height (4 cm). Medium obstacle height (11 cm). High obstacle height (16 cm).	2: Straight ahead walking without obstacle = flat ground surface without obstacle. Straight ahead walking and stepping over an obstacle height of 30% of leg length.	5: No obstacle = flat ground surface without obstacles (baseline initial and final trials). Low obstacle height (4 cm). Medium obstacle height (8 cm). High obstacle height (16 cm).	5: No obstacle = flat ground surface without obstacles (baseline initial and final trials). Low obstacle height (4 cm). Medium obstacle height (8 cm). High obstacle height (16 cm).
Obstacle condition order	Initial and final overground trials. Obstacle conditions were counterbalanced.	Not reported.	Two-step protocol without obstacle. Two-step protocol with obstacle.	Initial and final overground trials Obstacle conditions were randomised.	Initial and final overground trials Obstacle conditions were counterbalanced.
Number of trials per condition	5 for obstacle-crossing trials. 1 for baseline and final trials (no obstacle condition).	5.	5 valid trials. ^[y]^	5.	5.
Walking speed	Self-selected pace.	Self-selected pace.	Self-selected pace.	Self-selected pace.	Self-selected pace.
Experimental condition	Single task.	Single task.	Single task.	Single task.	Single task.
Practice	Not reported.	3 practice trials.	10 min familiarisation period.	Not reported.	Not reported.

^[y]^: Whenever a participant trod on the pressure platform, if foot contact was incomplete or the coefficient of variation of the duration of contact was greater than 4%, the trial was discarded. Note that all studies included single-task conditions and self-selected walking speed instruction and had the same number of experimental session and trials per obstacle crossing conditions.

**Table 6:** Gait parameters measured and data acquisition methods.

Author	Gill and Hung [19]	Gill and Hung [20]	Silva et al. [21]	Gill [22]	Desrochers et al. [18]
Parameters measured	Kinematics: Time from foot lift to maximum knee height, time from maximum knee height to foot contact, and maximum knee height. Ankle angles at foot contact to determine neutral (angles near 90 degrees) or dorsiflexed ankle positions (angles < 90 degrees) and sagittal knee and ankle angles at maximum knee height. Estimated variability of ankle positions at foot contact by calculating the coefficient of variation.	Kinematics: Maximum toe height for each leg during obstacle crossing; hip and knee angles in sagittal and frontal planes; vertical, anterior/posterior, and medial/lateral acceleration of centre of mass.	Kinematics: Plantar pressure parameters: relative foot temporal data for the initial contact, final contact, and duration of contact.	Kinematics: Velocity, percentage of gait cycle spent in swing and in stance, and coefficient of variation for each variable to obtain a measure of variability.	Kinematics: Gait velocity, step length and width, cadence, and single- and double-limb support during leading leg step across the obstacle.
		Kinetics: Ground reaction force: normalised vertical, anterior/posterior, and medial/lateral ground reaction forces of both legs.			
Acquisition system	Vicon Nexus Model 1.4 motion capture system (120 Hz) and 41 reflective markers.	Whole-body plug-in-gait model of Vicon Nexus 1.51 (120 Hz) with 7 infrared cameras and 41 reflective markers.	Two Footscan platforms (100 × 40 cm, 8192 sensors; RSscan International, Olen, Belgium; 250 Hz).	Gait carpet (Protokinetics, LLC; Peekskill, NY, USA; 610 cm long × 89 cm wide; 120 Hz).	-GAITRite software and custom Matlab scripts (Mathworks, Inc., Natick, MA, USA) (488 cm long × 61 cm wide; 120 Hz).
		Two AMTI OR6-6 force platforms (46 × 50 cm; 1200 Hz).			

**Table 7:** The influence of overweight/obesity on gait parameters during obstacle crossing.

Gait Parameters	Number of Studies	Study	Between-Group Differences	Results (Mean ± SD or Median (IQR)) and Direction of Difference (↑↓)
**Kinematics outcomes**				
Velocity (cm/s)	2	Gill, 2019	**NW vs. OBI: *p* = 0.03** **NW vs. OBII: *p* = 0.000002** **NW vs. OBIII: *p* = 0.000000058** **OBI vs. OBII: *p* = 0.018** **OBI vs. OBIII: *p* = 0.001**	**NW = 125.08 ± 1.98** **OBI = 105.72 ± 3.08 (↓ 15.5% vs. NW)** **OBII = 91.46 ± 3.06 (↓ 26.9% vs. NW; ↓ 13.5% vs. OBI)** **OBIII = 85.46 ± 2.43 (↓ 31.7% vs. NW; ↓ 19.2% vs. OBI).**
		Desrochers et al., 2021	***p* = 0.0001 (OB vs. C)**	**NW = 97.49 ± 6.14** **OB = 67.53 ± 5.83 (↓ 30.7% vs. NW)**
Leading-leg step length (cm)	1	Desrochers et al., 2021	**NW vs. OB: *p* = 0.01**	**NW = 60.40 ± 0.37** **OB = 51.94 ± 0.18 (↓ 14.0% vs. NW)**
Leading-leg step width (cm)	1	Desrochers et al., 2021	**NW vs. OB: *p* = 0.01**	**NW = 8.40 ± 0.21** **OB = 11.54 ± 0.70 (↑ 37.4% vs. NW)**
Leading-leg single-limb support (% of gait cycle)	1	Desrochers et al., 2021	**NW vs. OB: *p* = 0.001**	**NW = 41.38 ± 1.16** **OB = 38.87 ± 1.03 (↓ 6.1% vs. NW)**
Double-limb support (% of gait cycle)	1	Desrochers et al., 2021	**NW vs. OB: *p* = 0.001**	**NW = 8.90 ± 0.85** **OB = 11.09 ± 0.96 (↑ 24.5% vs. NW)**
Cadence (steps/min)	1	Desrochers et al., 2021	**NW vs. OB: *p* = 0.001**	**NW = 104.46 ± 2.33** **OB = 90.24 ± 3.07 (↓ 13.6% vs. NW)**
Stance time (% of gait cycle)	1	Gill, 2019	**NW vs. OBI: *p* = 0.012** **OBI vs. OBII: *p* = 0.029**	**NW = 61.64 ± 1.71** **OBI = 65.13 ± 0.35 (↑ 5.7% vs. NW; ↑ 4.6% vs. OBII)** **OBII = 62.83 ± 0.02** **Low obstacle (4 cm):** **NW = 60.79 ± 6.97** **OBI = 64.85 ± 2.31 (↑ 6.7% vs. NW)** **Medium obstacle (8 cm):** **NW = 60.52 ± 8.04** **OBI = 65.01 ± 3.71 (↑ 7.4% vs. NW)**
			**NW vs. OBI, low obstacle: *p* = 0.004**	
			**NW vs. OBI, medium obstacle: *p* = 0.007**	
Swing time (% of gait cycle)	1	Gill, 2019	**NW vs. OBI: *p* = 0.003** **NW vs. OBII: *p* = 0.001** **NW vs. OBIII: *p* = 0.00024**	**C = 42.55 ± 0.85** **OBI = 38.42 ± 0.85 (↓ 9.7% vs. NW)** **OBII = 37.72 ± 0.02 (↓ 11.4% vs. NW)** **OBIII = 37.14 ± 0.21 (↓ 12.7% vs. NW)**
CV velocity	1	Gill, 2019	**NW vs. OBII: *p* = 0.00042** **OBI vs. OBII: *p* = 0.00015** **NW vs. OBIII: *p* = 0.000023** **OBI vs. OBIII: *p* = 0.000006**	**NW = 0.03 ± 0.01** **OBI = 0.03 ± 0.01** **OBII = 0.06 ± 0 (↑ 80% vs. NW and OBI)** **OBIII = 0.07 ± 0.01 (↑ 100% vs. NW and OBI)**
CV stance time	1	Gill, 2019	NS	
CV swing time	1	Gill, 2019	NS	
Time from foot lift to maximum knee height (s)	1	Gill and Hung, 2012	**NW vs. OB, high obstacle: *p* = 0.04**	**High obstacle (16 cm):** **NW = 0.08 ± 0.01** **OB = 0.11 ± 0.01 (↑ 37.5% vs. NW)**
			NW vs. OB, low obstacle: *p* = 0.41	
			NW vs. OB, medium obstacle: *p* = 0.31	
Time from maximum knee height to foot contact (s)	1	Gill and Hung, 2012	**NW vs. OB, high obstacle: *p* = 0.04**	**High obstacle (16 cm):** **NW = 0.0011 ± 0.001** **OB = 0.0006 ± 0.0005 (↓ 45.5% vs. NW)**
			NW vs. OB, low obstacle: *p* = 0.13	
			NW vs. OB, medium obstacle: *p* = 0.96	
Maximum knee height	1	Gill and Hung, 2012	C vs. OB: *p* = 0.57	
Ankle angle at foot contact (°)	1	Gill and Hung, 2012	**C vs. OB, low obstacle: *p* = 0.04**	**Low obstacle (4 cm):** **NW = 99.24 ± 6.18** **OB = 79.90 ± 6.18 (↓ 19.5% vs. NW)**
			NW vs. OB, medium obstacle: *p* = 0.07	
			NW vs. OB, high obstacle: *p* = 0.89	
Sagittal ankle angle at maximum knee height (°)	1	Gill and Hung, 2012	NW vs. OB: *p* = 0.65	
Sagittal knee angle at maximum knee height (°)	1	Gill and Hung, 2012	NW vs. OB: *p* = 0.18	
Leading-leg toe clearance (cm)	1	Gill and Hung, 2014	NS	
Trailing-leg toe clearance (cm)	1	Gill and Hung, 2014	**NW, high low obstacle: *p* = 0.02**	**Low obstacle (4 cm):** **NW = 13.73 ± 1.47** **O = 23.13 ± 1.53 (↑ 38% vs. high obstacle)**
			**OB, low vs. high obstacle: *p* = 0.01**	**High obstacle (16 cm):** **NW = 18.38 ± 1.53 (↑ 33.9% vs. low obstacle)** **OB = 16.76 ± 1.59**
Leading-leg hip angles at maximum knee height in frontal plane (°)	1	Gill and Hung, 2014	**OB, high vs. medium and low obstacles: *p* = 0.02**	**Low obstacle (4 cm):** **OB = 47.28 ± 0.47**
				**Medium obstacle (11 cm):** **OB = 50.28 ± 0.52**
			No other effects were observed	**High obstacle (16 cm):** **OB = 66.75 ± 0.57 (↑ 32.8% and 41.18% vs. medium and low obstacles, respectively)**
Trailing-leg hip angles at maximum knee height in frontal plane (°)	1	Gill and Hung, 2014	No BMI or BMI*condition effects were observed (*p* > 0.05).	
Hip angles at maximum knee height in sagittal plane (°)	1	Gill and Hung, 2014	No BMI or BMI*condition effects were observed for both legs (*p* > 0.05).	
Trailing-leg knee angles at maximum knee height in sagittal plane (°)	1	Gill and Hung, 2014	**NW vs. OB, high obstacle: *p* = 0.04**	**Low obstacle (4 cm):** **NW = 6.99 ± 0.66** **OB = 11.09 ± 1.16 (↑ 58.7% vs. NW)**
			**NW vs. OB, low obstacle: *p* = 0.04**	**High obstacle (16 cm):** **NW = 4.64 ± 0.64** **OB = 12.02 ± 1.05 (↑ 159.1% vs. NW)**
			No other effects were observed.	
COM acceleration in anterior/posterior direction at maximum knee height of leading leg during crossing	1	Gill and Hung, 2014	**NW vs. OB, low obstacle: *p* = 0.006** No other effects were observed.	**Low obstacle (4 cm):** **C = 0.63 ± 0.08** **OB = 1.05 ± 0.05 (↑ 66.7% vs. NW)**
COM acceleration in anterior/posterior direction at maximum knee height of trailing leg during crossing	1	Gill and Hung, 2014	**NW vs. OB, low obstacle: *p* = 0.01**	**Low obstacle (4 cm):** **NW = 0.66 ± 0.12** **OB = 1.02 ± 0.12 (↑ 54.5% vs. NW; ↑ 45.7% and 82.1% vs. medium and high obstacles, respectively)**
				**Medium obstacle (11 cm):** **NW = 0.83 ± 0.06** **OB = 0.70 ± 0.06**
			**OB, low vs. medium and high obstacles: *p* = 0.01**	High obstacle (16 cm): NW = 0.73 ± 0.09 OB = 0.56 ± 0.09
COM acceleration in medial/lateral direction at maximum knee height	1	Gill and Hung, 2014	No BMI or BMI*condition effects were observed for both legs (*p* > 0.05).	
COM acceleration in vertical direction at maximum knee height	1	Gill and Hung, 2014	No BMI or BMI*condition effects were observed for both legs (*p* > 0.05).	
Duration of contact of HL, HM, MF, M5, M4, M3, M2, M1, T2-5, T1 of leading and trailing leg (ms)	1	Silva et al., 2018	No difference between OB and C for any area for both legs.	
**Kinetics outcomes**				
Trailing-leg normalised, anterior/posterior ground reaction forces at maximum knee height of leading leg during crossing (N/kg)	1	Gill and Hung, 2014	**OB, low vs. medium and high obstacles: *p* = 0.001**	**Low obstacle (4 cm):** **NW = 0.17 ± 0.06** **OB = 0.66 ± 0.02 (↑266.7%, and 100% vs. medium and high obstacles, respectively)**
			**NW, high vs. medium and low obstacles: *p* = 0.001**	**Medium obstacle (11 cm):** **NW = 0.29 ± 0.03** **OB = 0.18 ± 0.08**
			No other effects were observed.	**High obstacle (16 cm):** **NW = 0.33 ± 0.04 (↑ 94,1%, and 13.8% vs. low and medium obstacles, respectively)** **OB = 0.33 ± 0.12**
Leading-leg normalised, anterior/posterior ground reaction forces at maximum knee height of trailing leg during crossing (N/kg)	1	Gill and Hung, 2014	No BMI or BMI*condition effects were observed (*p* > 0.05).	
Trailing-leg normalised, medial/lateral ground reaction forces at maximum knee height of leading leg during crossing (N/kg)	1	Gill and Hung, 2014	**OB, high vs. medium obstacle: *p* = 0.01**	**Medium obstacle (11 cm):** **NW = 0.19 ± 0.06** **OB = 0.04 ± 0.02**
			No other effects were observed.	**High obstacle (16 cm):** **NW = 0.12 ± 0.07** **OB = 0.19 ± 0.05 (↑ 375% vs. medium obstacle)**
Leading-leg normalised, medial/lateral ground reaction forces at maximum knee height of trailing leg during crossing (N/kg)	1	Gill and Hung, 2014	No BMI or BMI*condition effects were observed (*p* > 0.05).	
Trailing-leg normalised, vertical ground reaction forces at maximum knee height of leading leg during crossing (N/kg)	1	Gill and Hung, 2014	**OB, high vs. medium obstacle: *p* = 0.05**	**Low obstacle (4 cm):** **NW = 1.38 ± 0.27** **OB = 1.99 ± 0.19**
			**NW, medium vs. low obstacle: *p* = 0.05**	**Medium obstacle (11 cm):** **NW = 2.14 ± 0.22 (↑ 55.1% vs. low obstacle)** **OB = 1.58 ± 0.29**
			No other effects were observed.	**High obstacle (16 cm):** **NW = 1.92 ± 0.26** **OB = 2.23 ± 0.24 (↑ 41.1% vs. medium obstacle)**
Leading-leg normalised, vertical ground reaction forces at maximum knee height of leading leg during crossing (N/kg)	1	Gill and Hung, 2014	No BMI or BMI*condition effects were observed (*p* > 0.05).	

CV: coefficient of variation; COM: centre of mass; HM: medial heel; HL: lateral heel; M1-M5: metatarsal areas, MF: midfoot; NS: non-significant differences; NW: normal-weight group; OB: overweight/obese group; OBI: Class I obesity group; OBII: Class II obesity group; OBIII: Class III obesity group; T1: hallux; T2-5: toes.* No BMI effect nor interaction between BMI and condition were observed. All five studies investigated kinematic parameters during obstacle crossing [18,19,20,21,22].

## Data Availability

No new data was created.

## Supplementary Materials

The following supporting information can be downloaded at: https://www.mdpi.com/article/10.3390/ijerph20115931/s1, File S1: PRISMA checklist. Ref [50] is cited in the Supplementary Materials.
